# 1590. Resistance to Etravirine, Doravine and Rilpivirine in People Living with HIV Experiencing Virologic Failure to a First-line Antiretroviral Regimen Based on a Non-Nucleoside Reverse Transcriptase Inhibitor (NNRTI) in a Latin American Country.

**DOI:** 10.1093/ofid/ofad500.1425

**Published:** 2023-11-27

**Authors:** Verónica Mingrone, Eliana Loiza, Norma Porteiro, Jamile Ballivian, Ezequiel Córdova

**Affiliations:** Fundación IDEAA, CABA, Ciudad Autonoma de Buenos Aires, Argentina; Fundación IDEAA, CABA, Ciudad Autonoma de Buenos Aires, Argentina; Fundación IDEAA, CABA, Ciudad Autonoma de Buenos Aires, Argentina; IECS, CABA, Ciudad Autonoma de Buenos Aires, Argentina; Fundación IDEAA, CABA, Ciudad Autonoma de Buenos Aires, Argentina

## Abstract

**Background:**

To date, etravirine (ETR) is the only NNRTI indicated in people living with HIV (PLHIV) after virologic failure (VF) to first-generation NNRTIs. Data about susceptibility to ETR, doravirine (DOR) and rilpivirine (RPV) after VF in PLHIV from Latin American countries are scarce. The aim of this study is to determine the prevalence of resistance to ETR, DOR, and RPV in PLHIV who experienced VF to a first-line NNRTI-based regimen, and factors associated with high-level resistance (HLR) to ETR.

**Methods:**

Retrospective cohort study. Prevalence of resistance to ETR, DOR and RPV was assessed by analyzing genotyping tests of adult PLHIV who experienced VF to a first-line NNRTI-based regimen, in Buenos Aires, Argentina (2010 to 2020). Stanford HIVdb v9.4 was used to interpret resistance profiles. We defined resistance as both intermediate resistance (IR) and HLR interpretations. A sample size of 125 subjects was calculated to allow estimation of the primary endpoint with sufficient precision (95%CI). A cross-sectional analysis was carried out to identify risk factors associated with HLR to ETR.

**Results:**

N=125; 81.6% were male. Median age at VF was 39.5 years (IQR: 34.0-35.0); 82,4% received efavirenz and 17.6% nevirapine. Duration of VF: 245 days (IQR 133-413). Median HIV-1 viral load (VL): 11255 c/mL (3606-45100); median CD4+ cell count: 192 cell/uL (86-333). Seventy-seven samples (61.6%) had ETR resistance-associated mutations (RAMs); 25 (20%) had mutations associated with the highest levels of reduced susceptibility (100I, 101P, 181I/V). Most frequent ETR RAMS were: 100I (16%), 190A (16%) and 181C (12%); DOR RAMs: 100I (16%), 188L (8.8%) and 106M (4.8%), and RPV RAMs: 100I (16%), 181C (11.2%) and 138A (7.2%). Prevalence of resistance to ETR was 44.8% (IR: 32.8%, HLR: 12%), resistance to DOR: 64% (IR: 45.6%, HLR: 18.4%) and to RPV: 52% (IR: 7.2%, HLR: 44.8%). ETR maintained susceptibility to DOR resistant strains in 27.2% of the cases. We found a statistically significant association between HIV-1 VL >10000 c/mL and the risk of developing HLR to ETR (X^2^=4.5, p=0.034).

**Conclusion:**

In our cohort, resistance to ETR after VF to a NNRTI-containing regimen was lower than to DOR and RPV. HLR was uncommon and associated with high HIV-1 VL. Almost one third of DOR-resistant strains remained susceptible to ETR.

**Disclosures:**

**Verónica Mingrone**, GSK/ViiV: Educational Courses|Janssen Pharmaceutical Companies: Educational Courses **Eliana Loiza, n/a**, GSK/ViiV: Educational Courses|Janssen Pharmaceutical Companies: Educational Courses **Norma Porteiro, n/a**, GSK/ViiV: Advisor/Consultant|GSK/ViiV: Grant/Research Support|GSK/ViiV: Educational Courses|Janssen Pharmaceutical Companies: Advisor/Consultant|Janssen Pharmaceutical Companies: Grant/Research Support|Janssen Pharmaceutical Companies: Educational Courses **Ezequiel Córdova, n/a**, GSK/ViiV: Advisor/Consultant|GSK/ViiV: Grant/Research Support|GSK/ViiV: Educational Courses|Janssen Pharmaceutical Companies: Advisor/Consultant|Janssen Pharmaceutical Companies: Grant/Research Support|Janssen Pharmaceutical Companies: Educational Courses

